# Estimating yield in commercial wheat cultivars using the best predictors of powdery mildew and rust diseases

**DOI:** 10.3389/fpls.2022.1056143

**Published:** 2022-12-15

**Authors:** Bita Naseri

**Affiliations:** Plant Protection Research Department, Kermanshah Agricultural and Natural Resources Research and Education Center, AREEO, Kermanshah, Iran

**Keywords:** cereals, leaf rust, stem rust, stripe rust, disease progress curve

## Abstract

**Introduction:**

This four-year research determined the best predictors of black, brown and yellow rusts and powdery mildew development in different wheat cultivars and planting dates across 282 experimental field plots.

**Methods:**

Parameters estimated by exponential (for black rust and powdery mildew) and Gaussian (for brown and yellow rusts) models, area under disease progress curve (AUDPC), and maximum disease severity were considered as disease progress curve elements. Factor analysis determined the most predictive variables among 19 indicators in order to describe wheat yield.

**Results:**

According to principal component analysis (PCA), 11 selected wheat diseases and yield predicators accounted for 60% of total variance in datasets. This PCA test described four principal components involving these selected predictors. Next, multivariate regression model, which developed according to four independent principal components, justified a noticeable part of yield variability over and within growing seasons.

**Discussion:**

Present findings may improve accuracy of future studies to examine seasonal patterns of powdery mildew and rusts, predict wheat yield and develop integrative disease management programs.

## Introduction

Wheat yellow rust caused by *Puccinia striiformis* Westlend f. sp. *tritici* Eriksson, brown rust by *Puccinia recondita* f. sp. *tritici*, and powdery mildew by *Blumeria graminis* f. sp. *tritici* Em. Marchal have been known as destructive diseases of wheat worldwide. The fast late season spread of wheat black rust epidemics, caused by *Puccinia graminis* Pers.:Pers. f. sp. *tritici* Erikss. & E. Henn., developed three weeks before harvest, can reduce wheat yield remarkably (Naseri, 2022). The progression of powdery mildew and rust diseases in susceptible cultivars could be accelerated by suitable agro-ecological conditions, as reported previously (Naseri and Marefat, 2019; Naseri and Sasani, 2020; Naseri and Sabeti, 2021; Naseri and Sheikholeslami, 2021a). For instance, plot-scale findings in Iran predicted the severity of black rust based on the timing of disease onset and cultivar maturity, the monthly mean minimum temperature and number of rainy days in spring, the number of days with minimum temperatures within 5–20°C and maximum relative humidity above 60% during autumn–winter–spring, wheat resistance, and sowing time (Naseri and Sabeti, 2021). Therefore, such influential agricultural and environmental predictors can be used to estimate the intensity of wheat rust and powdery mildew epidemics for sustainable disease management purposes. However, a joint estimation of wheat yield reductions due to these four major diseases is still missing.

To estimate yield reductions in wheat crops infected with rust and powdery mildew, an updated understanding of specific disease progression curve elements for these complex pathosystems appears to be crucial. To meet this requirement, the easy-to-use variables describing seasonal disease development for each pathosystem—black rust (Naseri, 2022), brown rust (Naseri and Jalilian, 2021), yellow rust (Naseri and Kazemi, 2020), and powdery mildew (Naseri and Sheikholeslami, 2021b)—have been previously characterized. These characterizations were provided according to the examination of standard models to determine the best predictors for the progression of wheat rusts and powdery mildew. Most of the previous studies estimated yield losses due to wheat rusts based on detecting general disease descriptors such as the area under the disease progress curve (AUDPC; Mideksa et al., 2018), the final disease severity, the coefficient of infection (Hei et al., 2015), the infection rate (Savary et al., 2015), and the latent period (Soko et al., 2018). However, none of them used specific disease curve elements to estimate wheat losses due to rust and powdery mildew. Therefore, a more accurate and up-to-date estimation of wheat yield based on multiple disease measurements over the growing season using specific disease-progress-curve elements is needed for powdery mildew and rust pathosystems.

Furthermore, detecting the magnitude of relationships among such specific mildew and rust progress curve elements appears to be an essential prerequisite for easier and more accurate estimating yield losses in wheat crops. Luo (2008) demonstrated that principal component analysis (PCA) can minimize problems associated with variable selection when fitting multivariate regression models involving many independent variables. Furthermore, it is also believed that a wider variability in the disease datasets following effective changes in agro-ecological features, in particular cultivar resistance, maturity time, planting date, and climatic conditions over growing seasons improves the descriptive value of disease predictors (Kranz, 2003; Naseri and Kazemi, 2020; Naseri and Sheikholeslami, 2021b). Thus, the current study aimed to: (i) evaluate jointly the predictive values of powdery mildew and rust progress curve elements; (ii) assess relationships between considered disease progress curves; and (iii) predict yield according to powdery mildew and rust progress descriptors developed for commercial wheat cultivars differing in the disease-resistance levels and the timing of maturity and planting.

## Materials and methods

### Experiment design and data collection

From the 2013–2014 season to 2016–2017, the progression of powdery mildew and yellow, brown, and black rust diseases in winter wheat was assessed and modeled across experimental field plots established at the research station (latitude 34˚7´ north, longitude 46˚28´ east) of Kermanshah, as characterized in **Table 1**. Field plots were prepared based on a split-plot design with three replicates per treatment. To improve variability in the progression of powdery mildew and rust diseases across the experimental plots, various planting dates and wheat cultivars were involved in this four-season research. The bread wheat cultivars had been registered by the Seed and Plant Improvement Institute, Karaj, Iran, as reported earlier (Naseri and Kazemi, 2020). The following classification was considered according to the maximum disease severity ratings reported earlier: (1) Bahar semi-resistant to brown rust and susceptible to black and yellow rust; (2) Baharan resistant to black rust, semi-resistant to brown rust and powdery mildew, and susceptible to yellow rust; (3) Chamran II resistant to powdery mildew and susceptible to black, brown, and yellow rust; (4) Parsi resistant to brown rust and susceptible to powdery mildew, black and yellow rust; (5) Pishgam semi-resistant to black, brown, and yellow rust, and susceptible to powdery mildew; (6) Pishtaz resistant to black and brown rust, and susceptible to powdery mildew and yellow rust; (7) Sirwan resistant to brown rust, semi-resistant to black rust and powdery mildew, and susceptible to yellow rust; (8) Sivand semi-resistant to powdery mildew and susceptible to black, brown, and yellow rust.

**Table 1:** Experimental plots properties, factor categories and measurements.

Assessments	Year	Plot no.	Plot size (m^2^)	Experimental factors	
				Planting date (Main plot)	Cultivar (Subplot)
Black rust	2013–14	72	19.2	Oct. 10, Nov. 7, Dec. 3 and 31	Bahar
Brown rust	2014–15	63	24	Oct. 12, Nov. 14, Dec. 19	Baharan
Powdery mildew	2015–16	63	6	Oct. 27, Dec. 13 and 30	Chamran II
Yellow rust	2016–17	84	6	Oct. 11, Nov. 15, Dec. 11, Jan. 5	Parsi
Yield (kg/ha)					Pishgam Pishtaz
					Sirwan Sivand

Field plot properties:

–Irrigated by sprinkler system at 7–10 days intervals.

–Ploughed and fertilized with 225 kg/ha urea and 50 kg/ha superphosphate.

–Pesticide decis applied at 180 kg/ha.

–Lacking fungicide applications.

–Cool temperate climate with annual mean temperature and rainfall of 13.7°C and 479.8 mm.

**Figure 1:**
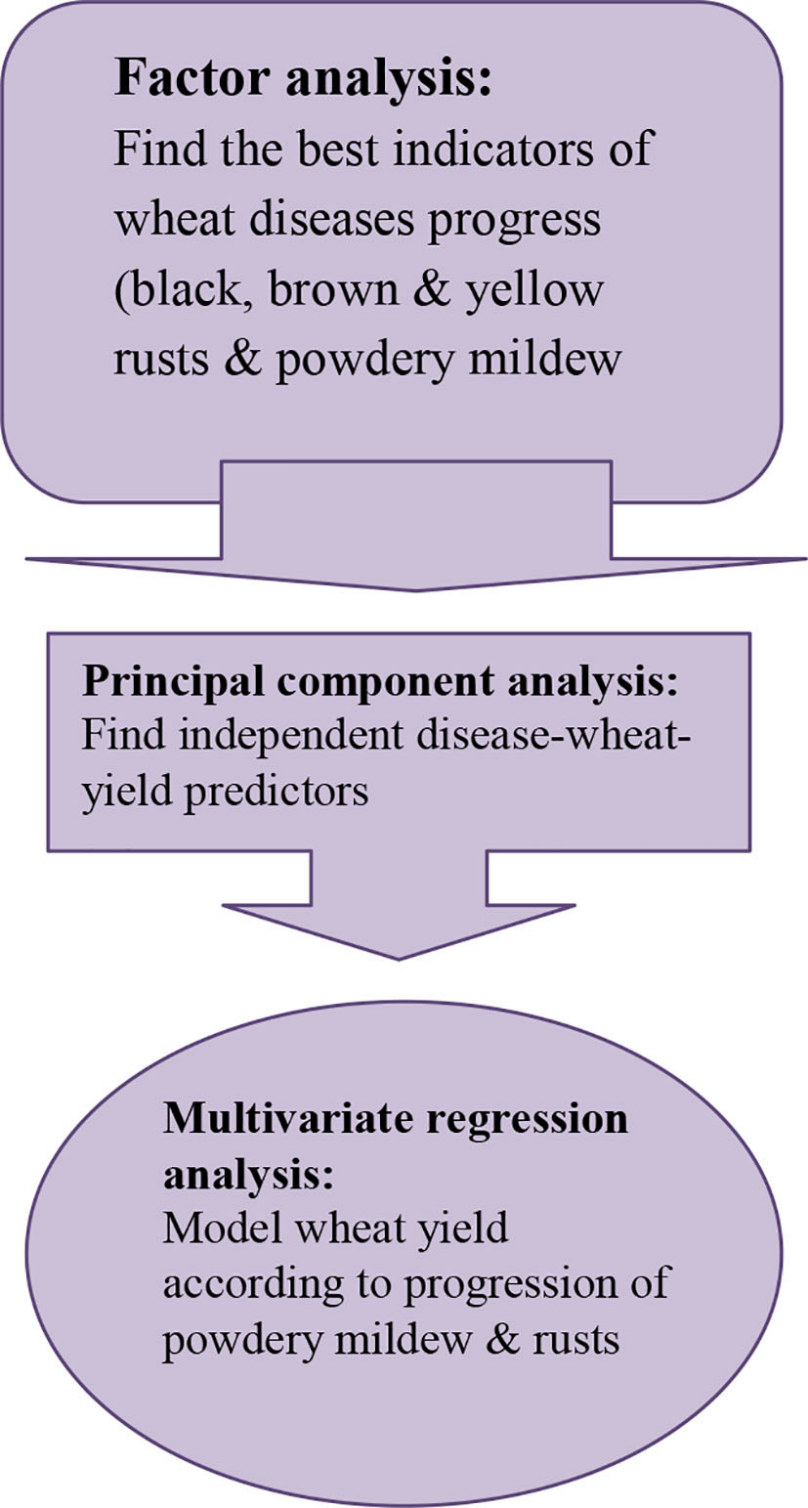
A flowchart demonstrating main study statistical procedures and outcomes.

According to the earlier reports, the severity of wheat diseases was rated every 7–10 days as the percentage of leaf area covered by black (Naseri and Sabeti, 2021), brown (Naseri and Sasani, 2020), and yellow (Naseri and Marefat, 2019) pustules for the three youngest leaves of three to five randomly inspected plants per plot. The percentage of leaf and stem area covered by whitish talcum-like symptoms of powdery mildew was recorded as the severity rating for at least three plants per plot (Naseri and Sheikholeslami, 2021a). The progression of these four major diseases of wheat across 282 field plots (72 plots in 2014, 63 plots in either 2015 or 2016, 84 plots in 2017) was characterized using the following disease progress curve elements: (1) the AUDPC based on the disease severity ratings over time; (2) maximum disease severity over the growing season; and (3) Gaussian and exponential curve parameters estimated based on the disease severity data as reported previously (Naseri and Kazemi, 2020; Naseri and Jalilian, 2021; Naseri and Sheikholeslami, 2021b; Naseri, 2022). All the statistical analyses were performed using GENSTAT (Payne et al., 2009), which fits standard curves based on the maximum likelihood (**Figure 1**). The exponential model was best fitted to the powdery mildew and stem rust severity data (**Table 2**). In this asymptotic regression model, *a* is described as the initial value or constant term, *b* is the increase factor, *r* is the increase rate, and x is the time interval (day) between two consecutive disease measurements. This model indicates a slow increase in disease at first, and then a rapid and more rapid increase without bounds. With *b >*1 the model demonstrates an exponential increase and with 0 < *b* < 1 an exponential decrease occurs. In the Gaussian models developed for brown and yellow rusts, a is the constant term, b is the height of the curve’s peak, m is the position of the center of the peak, s (the standard deviation) is the width of the Gaussian bell, and x is the time interval (**Table 2**).

**Table 2:** Models fitted to black, brown, and yellow rust, and powdery mildew progression datasets collected from wheat cultivars differing in planting dates.

Diseases	Standard models^a^	Parameters estimated
Black rust	a + br^x^	Exponential parameter *b*
		Exponential parameter *r*
Brown rust	a + b ∗ Gauss ((x − m)/s)	Gaussian parameter *b*
		Gaussian parameter *m*
		Gaussian parameter *s*
Powdery mildew	a + br^x^	Exponential parameter *b*
		Exponential parameter *r*
Yellow rust	a + b ∗ Gauss ((x − m)/s)	Gaussian parameter *b*
		Gaussian parameter *m*
		Gaussian parameter *s*

^a^Constant term which was ignored in estimating parameters; x, time intervals (days) between consecutive disease measurements.

A factor analysis (FA) was used to evaluate the associations of 19 descriptors determined for the progression of powdery mildew and rust and wheat yield with those principal factors that received eigenvalues (proportion of data variance) ≥1.0. Either of the principal factors provided a linear regression of the descriptors involved. To predict the productivity of wheat cultivars infected by powdery mildew and rusts, it was necessary to decrease the number of descriptors to minimize the problematic collinearity among the descriptors considered (loading values ≥0.35; Kranz, 2003) for regression modeling. In the next step, a principal component analysis (PCA) using a correlation matrix examined the contributions of powdery mildew, black, brown, and yellow rust, and wheat productivity descriptors in the principal components (PCs). This statistical tool simplified the selection of significant predictors (with loadings ≥0.35; Kranz, 2003) to develop regression models for estimating wheat yield. Briefly, the FA reduced the number of wheat diseases and yield predictors from 19 (**Table 3**) to 11 (**Table 4**) to minimize collinearity. Then, the PCA test examined the interrelationships among these 11 predictors considered for modeling. From the stepwise selection of predictors, the two criteria of the adjusted coefficient of determination (R^2^) and Mallows Cp were used (Brusco and Stahl, 2005; Kakhki et al., 2022). In addition, the graphical appraisal of normally distributed residuals, *F*-test, and R^2^ were used for the assessment of model fitness (Naseri and Sabeti, 2021).

**Table 3:** Factor analysis of black, brown, and yellow rust, and powdery mildew progression in wheat cultivars differing in sowing times.

Diseases	Parameters estimated	Factors				
		1	2	3	4	5
Black rust	Area under disease progress curve	0.27	0.21	0.06	0.33	−0.05
	Exponential parameter *b*	−0.08	0.13	−**0.46**	−0.28	−0.23
	Exponential parameter *r*	−0.06	−0.02	0.00	−0.07	**0.45**
	Maximum disease severity	0.27	−0.32	0.17	0.09	0.08
Brown rust	Area under disease progress curve	0.30	0.21	−0.05	0.01	0.25
	Gaussian parameter *b*	−0.01	0.11	0.11	−0.33	−**0.43**
	Gaussian parameter *m*	0.22	0.30	0.19	−0.30	−0.22
	Gaussian parameter *s*	0.19	**0.36**	0.17	−0.28	−0.07
	Maximum disease severity	0.31	0.27	0.04	0.05	0.18
Powdery mildew	Area under disease progress curve	0.21	−0.33	**0.42**	0.06	−0.06
	Exponential parameter *b*	−0.09	0.12	0.02	0.14	**0.37**
	Exponential parameter *r*	0.31	−0.31	−0.02	−0.07	−0.04
	Maximum disease severity	0.22	−0.34	**0.38**	0.02	−0.10
Yellow rust	Area under disease progress curve	0.32	−0.07	−0.34	−0.01	−0.01
	Gaussian parameter *b*	−0.02	−0.02	−0.14	0.34	−**0.40**
	Gaussian parameter *m*	0.31	−0.30	−0.13	−0.09	−0.02
	Gaussian parameter *s*	0.27	−0.17	−0.33	−0.09	0.16
	Maximum disease severity	0.32	−0.03	−0.31	0.08	−0.08
Yield (kg/ha)		0.02	0.18	−0.05	**0.59**	−0.27
Eigenvalues		6.32	2.76	1.69	1.22	1.08
Variation (%)		33.24	14.51	8.89	6.44	5.66
Accumulated variation (%)		33.24	47.75	56.64	63.08	68.74

a A bold number indicates a significant loading value ≥ 0.35.

**Table 4:** Principal component analysis of wheat production, black, brown, and yellow rust predictors selected based on factor analysis.

Variables		Principal components			
		1	2	3	4
Yellow rust	Area under disease progress curve	**0.44**	**0.44**	−0.01	0.09
Yellow rust	Maximum disease severity	**0.46**	**0.45**	0.08	0.05
Brown rust	Gaussian parameter *s*	0.17	0.28	−**0.40**	−0.27
Black rust	Exponential parameter *b*	−0.21	**0.44**	−0.14	0.21
Powdery mildew	Area under disease progress curve	**0.48**	−**0.39**	0.03	−0.04
Powdery mildew	Maximum disease severity	**0.49**	−**0.35**	0.02	−0.05
Yield (kg/ha)		−0.04	0.20	**0.46**	−0.32
Black rust	Exponential parameter *r*	−0.10	−0.10	−0.08	**0.47**
Powdery mildew	Exponential parameter *b*	−0.18	−0.05	0.13	−**0.42**
Brown rust	Gaussian parameter *b*	-0.05	−0.02	−**0.39**	−**0.60**
Yellow rust	Gaussian parameter *b*	0.00	0.11	**0.65**	-0.10
Eigenvalues		2.72	1.70	1.09	1.05
Variation (%)		24.74	15.45	9.88	9.52
Accumulated variation (%)		24.74	40.19	50.07	59.59

A bold number indicates a significant loading value ≥ 0.35.

## Results

### Factor analysis

From the FA, the five principal factors explained 69% of the variance in powdery mildew, black, brown, and yellow rust and productivity datasets collected from cultivars planted at different times over the four seasons of wheat growth (**Table 3**). The first principal factor accounted for 33% of the total data variance, evidencing the highest positive loading values for the contributions of AUDPC and maximum disease severity predictors of yellow rust progress on the wheat cultivars tested. Because of the non-significant associations (loading <0.35) of 19 disease and yield predictors with the first principal factor, the predictors with the highest loadings were considered for the remainder of the statistical analyses. Therefore, this factor of the FA was considered yellow rust progress factor. The Gaussian parameter *s* of brown rust contributed significantly to the second principal factor, justifying 15% of the total data variance. This suggested the second factor as the factor of brown rust progress in wheat.

The third principal factor, accounting for 9% of the data variance, provided a moderately negative loading for the exponential parameter *b* of black rust. It also provided moderately positive moderate loadings for the AUDPC and maximum disease severity predictors of powdery mildew development in wheat. This factor was defined as the factor of black rust and powdery mildew progress modeled by exponential curves. The fourth principal factor, explaining 6% of the data variance, provided a positively significant loading value for the contribution of the wheat yield predictor. This suggests that the fourth principal factor to be considered as the wheat productivity factor. The fifth principal factor justified 6% of the total data variance, representing the positive contributions of the exponential parameters *b* and *r* estimated for powdery mildew and black rust progress in wheat, respectively. This principal factor also provided negative loading values for the Gaussian parameter *b* estimated for the progression of brown and yellow rusts in those wheat cultivars studied during four growing seasons. Thus, the fifth principal factor was defined as the progression factor of all four major diseases in this crop (**Table 3**).

### Principal component analysis

From the PCA test, the four principal components justified 60% of the variance in black, brown, and yellow rust and powdery mildew progress curve elements described during the four growth seasons of eight commercial wheat cultivars differing in planting times (**Table 4**). The first principal component explained 25% of the total data variance. This PC provided the positively moderate loading values for the AUDPC and maximum disease severity of powdery mildew and yellow rust in wheat. The same powdery mildew and yellow rust predictors as the first PC were associated with the second PC, which accounted for 16% of the data variance. However, the AUDPC and maximum disease severity of powdery mildew provided negative contributions to the second principal component. In addition, the exponential parameter *b* estimated for wheat black rust progress was positively associated with the second principal component.

The third PC, explaining 10% of the data variance, determined the highest loading value (0.65) for the Gaussian parameter *b* estimated for the progression of yellow rust in wheat (**Table 4**). This principal component also provided the negatively moderate loadings for the Gaussian parameters, *b* and *s*, estimated for brown rust progress in wheat. The yield predictor was also positively associated with the third PC. Thus, this factor of the PCA test demonstrated the reverse linkage of the Gaussian parameters, *b* and *s*, estimated for brown rust progress to the yield predictor. The fourth principal component, which accounted for 10% of the total data variance, indicated the highly negative contribution of the Gaussian parameter *b* estimated for wheat brown rust. This PC also suggested the negative and positive correspondences of the exponential parameters *b* estimated for powdery mildew and *r* estimated for black rust progress in wheat crops, respectively. Therefore, according to the PCA results, the productivity in wheat was significantly associated with the progression of black, brown, and yellow rust and powdery mildew examined over the four growth seasons at the field-plot scale.

### Regression analysis

**Figure 2:**
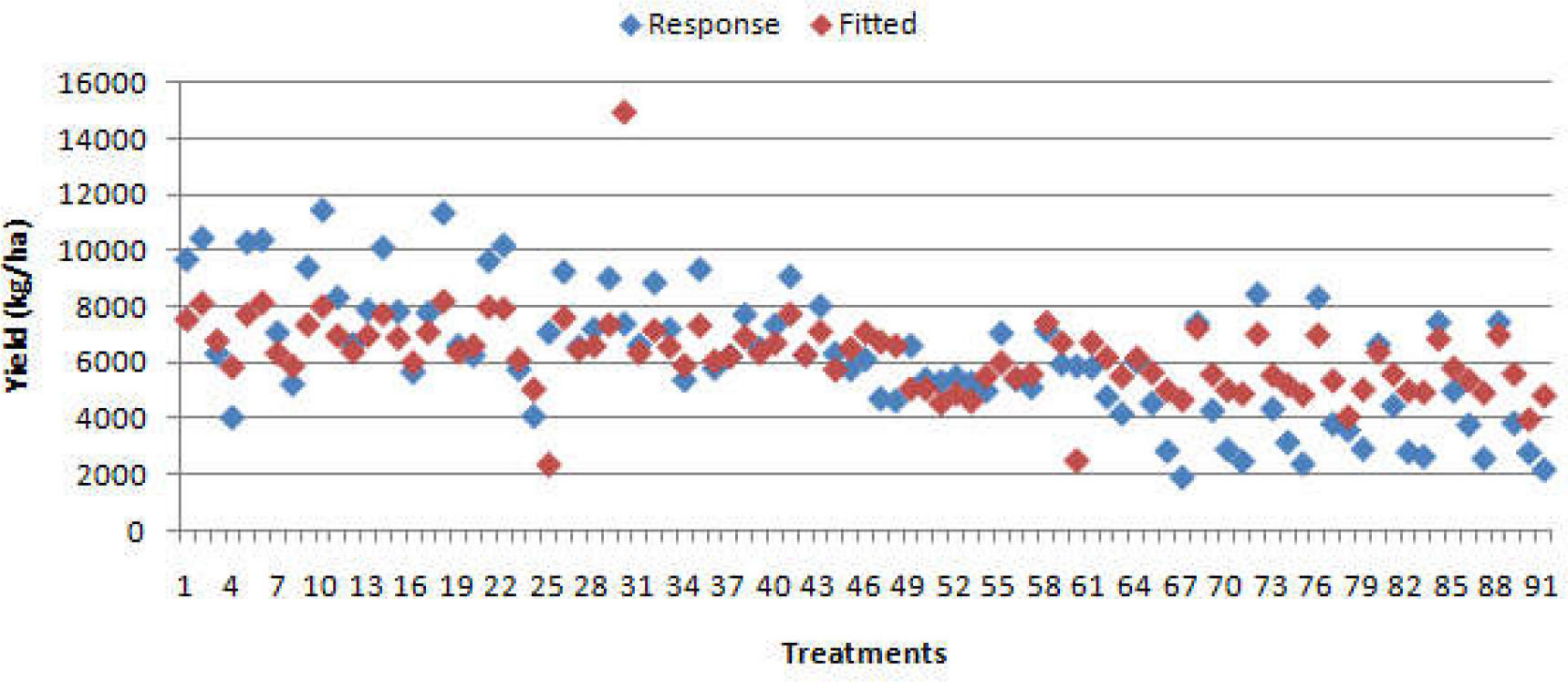
Simple regression analysis (*r* = 0.64; *F* probability = 0.001) of observed data against wheat yield data fitted by multivariate regression model; treatments refer to average values provided for three replicate plots per cultivar, planting date, and year.

The multivariate regression analysis justified 41% of the variability in yield (kg/ha; *F* probability = 0.001; R^2^ = 0.41) of the wheat cultivars treated with different planting dates across 282 field plots (72 in 2014, 63 in either 2015 or 2016, and 84 in 2017) according to the black, brown, and yellow rust and powdery mildew progress predictors (**Table 5**). The variables were selected for the regression model according to the independent principal components provided by the PCA test, followed by stepwise selection. The linear combinations of the following 11 predicators, AUDPC and maximum severity of powdery mildew and yellow rust, exponential parameters *b* and *r* estimated for black rust, exponential parameters *b* estimated for powdery mildew, Gaussian parameters, *b* and *s*, estimated for brown rust, Gaussian parameter *b* estimated for yellow rust, corresponded significantly with the yield (kg/ha) in the wheat cultivars characterized over the four growing seasons. Then based on a simple regression analysis, the observed data for wheat yield was regressed significantly to the fitted data (**Figure 2**). Therefore, the production of wheat was estimated according to the predictors developed for the over-season progression of wheat rust and powdery mildew examined at field-plot scale.

**Table 5:** Multiple regression analysis of wheat yield (kg/ha) according to principal component analysis of powdery mildew, black, brown, and yellow rust progress predictors (R^2^ = 41%).

Variables	Parameter estimates	Standard errors	*t*-probability
Constant	6219.00	189.00	< 0.001
PC1	-88.00	115.00	0.446
PC2	462.00	146.00	0.002
PC3	1065.00	183.00	< 0.001
PC4	-740.00	186.00	< 0.001

PC, Principal component.

## Discussion

The current research examined the specific predictors of wheat powdery mildew and rust to achieve more accurate and easy-to-use models for predicting wheat yield in the future. Considering reductions in the predictive value of regression-based models due to collinearity among disease predictors (Pietravalle et al., 2003), an up-to-date effort is needed to model wheat yield according to the best curve elements of wheat powdery mildew and rust progress. The progression of black, brown, and yellow rust and powdery mildew over a four-year study on eight commercial wheat cultivars with various levels of resistance and planting dates was compared according to factor analysis of 19 disease curve elements. Then, the best disease predictors were considered by performing the PCA procedure as recommended by Luo (2008) and Kranz (2003). The earlier recommendation from Kranz (2003) on the correspondence of a fitted progress curve to high variability in disease development patterns was considered in the present research. Therefore, the present experimental design improved not only the heterogeneity in powdery mildew and rust progress across the 282 field plots examined during the four growing seasons but also the variations in the yield levels determined for the eight commercial cultivars planted at different times.

Khan et al. (1997) modeled yield losses in wheat due to brown rust in eight cultivars at five locations in Mississippi over a 4-year period (1986–1989) according to a negative linear relationship between crop yield and disease development. Total grain yield was reduced by 1% for each 1% increase in rust pustules at flag leaf stage (Khan et al., 1997). However, the predictive values of other rust diseases and powdery mildew in conjunction with brown rust for wheat stem rust progress remain unknown. Furthermore, the recent findings advanced our understanding of specific disease progress curve elements described for black rust (Naseri, 2022), brown rust (Naseri and Jalilian, 2021), yellow rust (Naseri and Kazemi, 2020), and powdery mildew (Naseri and Sheikholeslami, 2021b) development in wheat crops. However, a better insight into yield estimations according to such specific disease progress predictors is still missing. To the best of our knowledge, this is the first report of the fitted multivariate regression model for predicting wheat yield based on specific and up-to-date disease progress curve elements described for powdery mildew and rust pathosystems. Moreover, this finding, which was achieved through highly diverse variations in disease progress curves and wheat yield levels, increased the descriptive value of the regression model as advised by Kranz (2003). The current importance of selected powdery mildew and rust progress predictors, which account for 41% of variance in wheat yield, underscores the priority of involving these disease curve elements in future studies on disease epidemiology, breeding slow-rusting resistant cultivars, and developing disease management methods. It should be noted that these 11 disease and yield predictors considered in the current study justified 60% of the data variance according to the PCA results. Thus, this work identified a manageable set of powdery mildew and rust progress curve elements to be used for more accurate yield estimation in wheat cropping systems. In the future, research may consider further influential agronomic and environmental predictors to improve variations in datasets and the contributions of disease progress curve elements to wheat productivity.

Previous studies characterized wheat rust progress using final disease severity, infection rate (Hei et al., 2015), latent period, and AUDPC (Kranz, 2003) for either disease or yield prediction purposes (Mideksa et al., 2018; Soko et al., 2018). However, none of them compared various powdery mildew and rust progress curve elements for their predictive value to estimate wheat yield. The current PCA results signified the relevance of the AUDPC and maximum disease severity as best predictors of powdery mildew and yellow rust progress when estimating wheat yield in commercial cultivars at experimental plot scale. However, these two disease progress predictors did not indicate a significant correlation for black and brown rusts in wheat. Such findings notify us that the AUDPC and maximum disease severity may not be as descriptive as the exponential or Gaussian parameters estimated for black and brown rusts, respectively. Thus, it is more reliable to consider the exponential and Gaussian parameters specified in the present study for more accurate black and brown rust measurements in the future. Therefore, this seems to be the first comparison of predictive values of disease progress curve elements between these four prevalent and destructive diseases of wheat. Indeed, further investigation is required to identify the physiological and molecular bases for such field observations.

As a conclusion, the specific disease predictors, which had been developed separately for each of the diseases (Naseri and Kazemi, 2020; Naseri and Jalilian, 2021; Naseri and Sheikholeslami, 2021b; Naseri, 2022), were used to predict wheat yield. Attempts were also made to improve the predictive values of powdery mildew and rust progress variables described according to highly diverse datasets on the progression of diseases. Such variations were provided for each crop–disease pathosystem following the treatment of different wheat cultivars with influential planting dates as documented previously (Naseri and Marefat, 2019; Naseri and Sasani, 2020; Naseri and Sabeti, 2021; Naseri and Sheikholeslami, 2021a). A total of 5,640 disease observations (282 plots + four diseases + five assessment times) were conducted to simulate the interaction of wheat yield (282 yield measurements) with these four major diseases under field conditions. Therefore, the current findings obtained by studying a manageable volume of experimentation might advance our understanding of the epidemiology of destructive wheat diseases from a sustainable production viewpoint.

## Data availability statement

The raw data supporting the conclusions of this article will be made available by the authors, without undue reservation.

## Author contributions

BN: Designing and performing the work, data collecting, interpreting the data, and writing the paper. The author confirms being the sole contributor of this work and has approved it for publication.
